# Differential expression of TIM-3 between primary and metastatic sites in renal cell carcinoma

**DOI:** 10.1186/s12885-019-5273-5

**Published:** 2019-01-10

**Authors:** Xingming Zhang, Xiaoxue Yin, Haoran Zhang, Guangxi Sun, Yaojing Yang, Junru Chen, Kunpeng Shu, Jinge Zhao, Peng Zhao, Ni Chen, Jia Wang, Pengfei Shen, Hao Zeng

**Affiliations:** 1 Department of Urology, West China Hospital, Sichuan University, No. 37 Guoxue Xiang, Chengdu, 610041 Sichuan China; 2 Institute of Urology, West China Hospital, Sichuan University, Chengdu, People’s Republic of China 610041; 3 Department of Pathology, West China Hospital, Sichuan University, Chengdu, People’s Republic of China 610041

**Keywords:** Differential expression, Metastases, Primary tumor, Renal cell carcinoma, TIM-3

## Abstract

**Background:**

Due to the significant heterogeneity of renal cell carcinoma (RCC), immune checkpoints may express differently between primary and metastatic tumor. We aimed to evaluate the differential expression of TIM-3 between the primary and metastatic sites of RCC.

**Methods:**

Cases of RCC with metastases resected or biopsied at West China Hospital between January 2009 and November 2016 were included. Clinicopathological parameters were retrospectively extracted. SPPS 22.0, GraphPad Prism 6 and R statistical software were applied for data analysis.

**Results:**

A total of 163 cases were included. Immunohistochemical results showed that the overall detection rate of TIM-3 was 56.4% (92/163). The detection rate of TIM-3 in the primary (53.0%, 44/83) was numerically higher than that of the metastasis (42.6%,79/174). Although the concordance rate of TIM-3 between the primary and metastasis was as high as 66.3% (55/83) in the paired cohort, a significant statistically difference of TIM-3 expression between the primary and metastasis was observed (χ2 = 4.664, *p* = 0.002), with a poor consistency (Kappa = 0.331, *p* = 0.002). Subsequent survival analysis suggested that TIM-3 expression either in the primary or metastatic tumor was associated with longer progression-free survival (PFS) (HR: 0.67, 95% CI 0.45–0.99, *P* = 0.02) and overall survival (OS) (HR: 0.52, 95% CI 0.33–0.82, *P* < 0.001). The expressions of TIM-3 in the primary, metastatic tumors and patients treated with targeted agents all played as favorable factors for PFS and OS. Further multivariate analysis showed that, in the whole cohort, TIM-3 expression in metastatic tumor increased the predicted accuracy (PA) of the whole model of PFS from 74.7 to 75.6% (*P* = 0.02). For OS, the PA of whole model was increased from 78.1 to 81.1% by adding TIM-3 expression in the metastasis (*P* = 0.005). The same trends were also observed in paired patients and patients treated with targeted agents. In conclusion, the expression difference between the primary and metastatic tumor of TIM-3 was significant. Biopsy or resection of the metastases may provide a more accurate biological information for clinician’s decision-making and the patient’s prognosis. What’s more, the role of TIM-3 in the RCC still remains controversy, further study are needed to verify the conclusion.

## Introduction

Of all patients with kidney cancer, 20–30% of them were diagnosed as metastatic renal cell carcinoma (RCC) at initial diagnosis, and about 20–40% of localized renal cell carcinoma would develop distant metastasis in spite of having been treated with radical nephrectomy [1]. All along kidney cancer was recognized as immunogenic tumors. Thus in the 1990s, non-specific immunotherapy with cytokines was seen as the standard treatment of mRCC. However, due to the low efficacy of cytokine drugs, the objective response rate was only 5–27%, and the median progression-free survival (PFS) was only 3–5 months, accompanied by evident side effects. Compared with cytokines, targeted agents showed significant tumor response and beneficial survival outcomes. Nevertheless, the median survival time was still only 8–30 months (according to different prognostic risk group) [2–4]. Therefore, development of more effective drugs to improve patients’ survival outcomes is needed.

Recent studies demonstrated that immune checkpoints have played key roles in the mechanism of immune escape [5–8]. In the tumor immunologic microenvironment, the most critical part of antitumor activity is the T cell activation. Activated T cells can effectively recognize and kill tumor cells. With the breakthroughs in molecular immunology, researchers have found the presence of a variety of co-stimulatory and co-inhibitory signal receptors—the two common regulation of T cell activation—on T cell surface [9]. The co-stimulatory signal receptor is like an “accelerator” to promote T cell activation, thereby facilitating the immune cells killing tumor cell. Co-inhibitory signal receptor is like a “brake” trigger leading to T cell inactivation by tumor cells using its immunological checkpoints to combine with co-inhibitory signal receptor. Ultimately, tumor cells survive, or rather the occurrence of immune escape [10].

Through analyzing the expression of immune checkpoints in tumor and the effect of checkpoints inhibitors, researchers found a positive correlation between immune checkpoints (such as PD-L1) over-expression and the treatment response in lung cancer and melanoma [11, 12]. However, there was no such correlation observed in RCC [13]. Due to the significant heterogeneity of RCC, immune checkpoints may express differently between primary and metastatic tumor. This differential expression suggest that the evaluation of expressions of immune checkpoints in metastasis might offer more accurate prediction of treatment response of immunotherapy [14, 15]. Several studies have shown that PD-L1 differentially expressed between the primary and metastasis in myeloma and RCC [14, 15]. However, it is unclear that whether TIM-3 (T cell immunoglobulin and mucin-domain containing-3), another immune checkpoint, is in the same situation with that of PD-L1 in RCC.

TIM-3 is an immune checkpoint (co-inhibitory signal receptor) located in T-cell [16]. Despite the functional role of Tim-3 was first described to negatively regulate the Th1 response, it seems to play a more complicated role in regulating anti-tumor response [16]. In fact, stimulation of Tim-3 is considered to act as both inhibitory and activating signal, which has been demonstrated in infectious diseases, autoimmune disorders and cancer immunity [17–20].

Therefore, in the present study, we aimed to compare the differential expression of TIM-3 in the primary and metastatic sites of RCC, and additionally, to find the role of TIM-3 in predicting patient’s prognosis.

## Materials and methods

### Patients and samples

Cases of RCC histologically diagnosed by focal resection or fine needle aspiration at West China Hospital from January 2009 to November 2016 were included. The inclusion criteria were metastatic disease at initial diagnosis with metastasis resected or biopsied, with or without companying specimens of the primary tumor. The exclusion criteria were metastases which were only suspected of deriving from kidney cancer, without screening of evidence of the primary tumor, or with a negative result in screening. All included cases were independently diagnosed by two urological pathologists (Ni Chen and Xiaoxue Yin), and the paraffin blocks of included cases were screened suitable for immunohistochemical staining.

Clinicopathological parameters were retrospectively extracted, including age, gender, metastatic sites, T stage, Eastern Cooperative Oncology Group (ECOG) status, International Metastatic Renal-Cell Carcinoma Database Consortium (IMDC) classification, ISUP grade, histological type, treatment, nephrectomy status and tumor necrosis. The end-points were PFS and OS. PFS was defined as the time from diagnosis to progression of disease or death. OS was defined as the time from diagnosis to all-cause death.

Expression of TIM-3 was detected by immunohistochemistry (IHC) by using anti-TIM-3 monoclonal antibody (Cell Signaling Technology, clone number: 45208S) at a 1:200 dilution. Positive signal of TIM-3 was on the cell membrane. Staining intensities were evaluated on a scale of 0(null), 1 + (weak), 2 + (moderate) and 3+ (strong) [14]. Positivity of TIM-3 was defined as the positive signal detected on > 5% tumor cells with staining intensity ≥1+. All staining results were independently assessed by two urological pathologists (Ni Chen and Xiaoxue Yin).

### Data analysis

SPPS 22.0, GraphPad Prism 6 and R statistical software were applied for data analysis. Continuous parameters were calculated as mean and SD and were analyzed by non-parametric test. Categorical parameters were calculated as proportions and were analyzed by chi-square test. Kappa test was applied to analyze the expression consistency between the primary and metastatic sites. The agreement was measured by Kappa coefficient: Kappa≤0.2 as indicating slight agreement, 0.4 < Kappa≤0.6 as fair, 0.6 < Kappa≤0.8 as substantial, > 0.8 as almost perfect agreement great. Kaplan-Meier and Cox’s proportional hazards regression model were applied to analyze PFS and OS. *P* < 0.05 was considered significant in all results. Predictive accuracy (PA) was calculated by using R software. Kaplan-Meier and Cox’s proportional hazards regression model were applied to analyze survival outcomes. Statistical significance was defined as *P* < 0.05. Predictive accuracy (PA) was calculated by using R software.

## Results

### Baseline characteristics of included patients

A total of 163 patients were included in this study from January, 2009 to November, 2016 in West China Hospital, Sichuan University. Patients’ characteristics were summarized in Table 1. There were 83 paired cases (both primary and metastatic tumors were available) and 80 cases of metastatic tumors with no accompanied primary tumors.

**Table 1:** Baseline clinicopathological parameters of included patients

	Paired (*N* = 83)	Metastatic (*N* = 80)	*P* value
Age, n (%)			0.546
< 70	76 (91.6)	71 (88.8)	
≥ 70	7 (8.4)	9 (11.2)	
Gender, n (%)			0.873
Male	54 (65.1)	53 (66.3)	
Female	29 (34.9)	27 (33.7)	
ISUP, n (%)			0.066
< 4	49 (67.1)	58 (80.6)	
≥ 4	24 (32.9)	14 (19.4)	
Histological Type, n (%)			0.032
ccRCC	59 (71.1)	68 (61.3)	
Non-ccRCC	24 (28.9)	12 (38.7)	
Pathology, n (%)			
Sarcomatoid	6 (19.4)	2 (28.6)	0.509
Necrosis	25 (80.6)	5 (71.4)	0.491
Nephrectomy, n (%)			0.001
Yes	80 (96.4)	57 (77.0)	
No	3 (3.6)	17 (23.0)	
ECOG, n (%)			0.801
0–1	53 (70.7)	59 (76.6)	
≥ 2	22 (29.3)	18 (23.4)	
IMDC, n (%)			0.293
Low	14 (18.3)	19 (28.4)	
Intermediate	43 (57.3)	37 (55.2)	
High	18 (24.0)	11 (16.4)	
T stage, n (%)			0.587
< 2b	46 (59.7)	12 (66.7)	
≥ 2b	31 (40.3)	6 (33.3)	
Metastasis, n (%)			
Lung	6 (8.1)	17 (28.8)	0.010
Brain	7 (9.5)	12 (20.3)	0.192
Liver	1 (1.4)	2 (3.4)	0.539
Bone	18 (24.3)	22 (37.3)	0.389
Lymph node	42 (56.8)	6 (10.2)	0.001
Treatment, n (%)			
Cytokine	14 (16.9)	8 (10.0)	0.345
Target therapy	34 (41.0)	30 (37.5)	0.882
Radiotherapy	7 (8.4)	7 (8.8)	0.673
Chemotherapy	3 (3.6)	5 (6.2)	0.360
Unknown	25 (30.1)	30 (37.5)	

### Expressions of TIM-3 in the whole and paired cohort

**Fig. 1:**
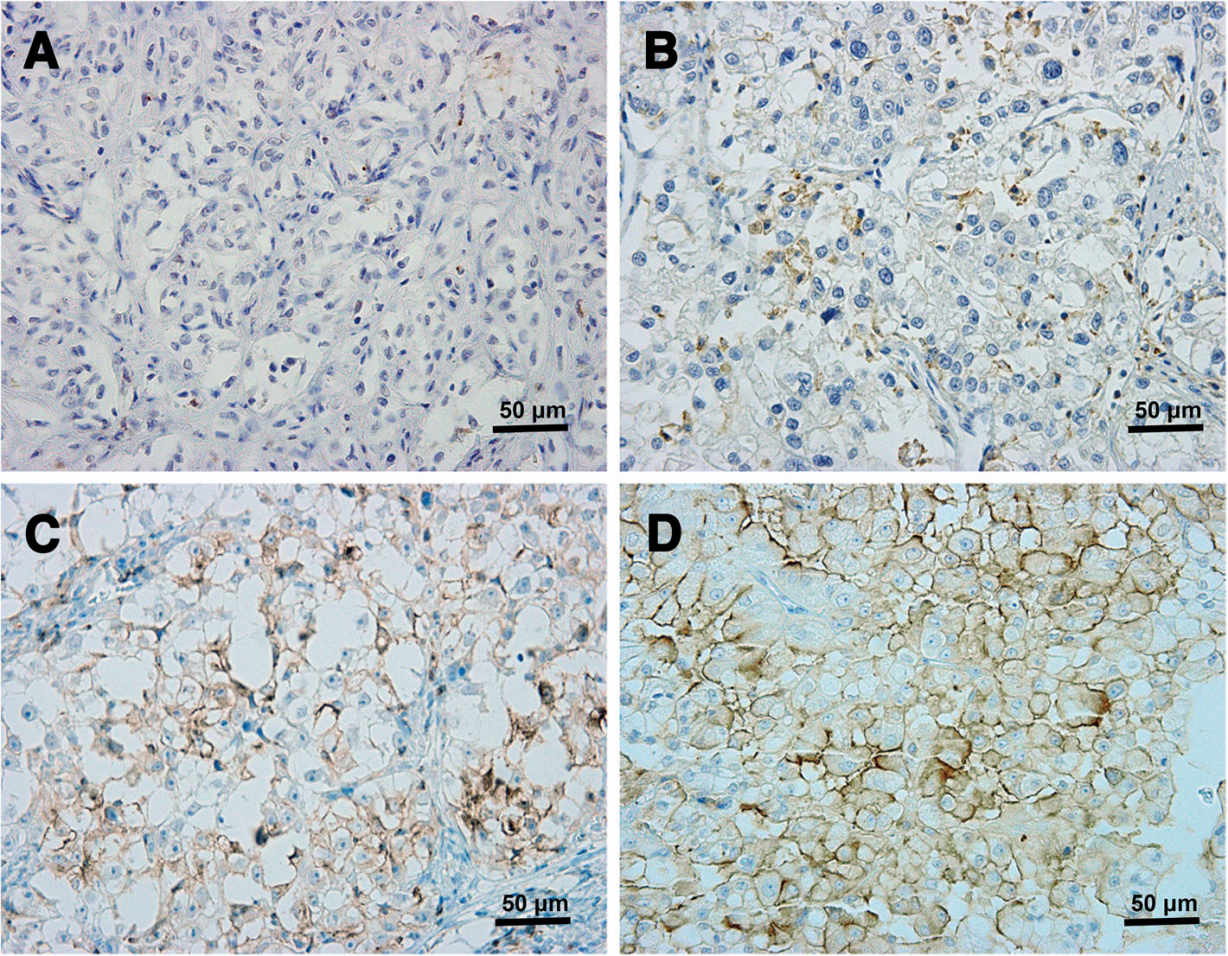
Representative images of immunohistochemical staining of TIM-3. **a** represented negative expression of TIM-3. **b**, **c** and **d** represented different positive intensities (1+, 2+ and 3+) of TIM-3 (original magnification: 200×)

Figure 1a showed negative expression of TIM-3 in RCC tumor. TIM-3 was mainly expressed on the membrane of tumor cells (Fig. 1b-d). In the whole cohort (*N* = 163), the overall detection rate of TIM-3 was 56.4%(92/163). The associations between clinical pathological parameters and TIM-3 expression in the primary or metastatic tumor were shown in Table 2. TIM-3 expression in patients with nephrectomy was higher that of patients without nephrectomy (χ2 = 4.96, *p* = 0.03). After stratified by IMDC classification, a gradually decreasing trend of the detection rates was observed from favorable (69.7%), intermediate (61.3) to high risk (37.9%) groups (χ2 = 7.01, *P* = 0.03).

**Table 2:** Relationship between the expression of TIM-3 and clinicopathological parameters

	TIM-3		*P* value
	Positive	Negative	
Total	92 (56.4)	71 (43.6)	
Age, n (%)			0.987
< 70y	83 (90.2)	64 (90.1)	
≥ 70y	9 (9.8)	7 (9.9)	
Gender, n (%)			0.259
Male	57 (53.3)	50 (46.7)	
Female	35 (62.5)	21 (37.5)	
ISUP, n (%)			0.055
< 4	67 (79.8)	40 (65.6)	
≥ 4	17 (20.2)	21 (34.4)	
Histological Type, n(%)			0.377
ccRCC	74 (58.3)	53 (41.7)	
Non-ccRCC	18 (50.0)	18 (50.0)	
Pathology, n (%)			
Sarcoma	5 (62.5)	3 (37.5)	0.898
Necrosis	20 (66.7)	10 (33.3)	0.958
Nephrectomy			0.026
Yes	84 (61.3)	53 (38.7)	
No	7 (35.0)	13 (65.0)	
ECOG, n (%)			0.239
0–1	68 (60.7)	44 (39.3)	
≥ 2	20 (50.0)	20 (50.0)	
IMDC, n (%)			0.030
Low	23 (69.7)	10 (30.3)	
Intermediate	49 (61.3)	31 (38.7)	
High	11 (37.9)	18 (62.1)	
T stage, n (%)			0.391
< 2b	38 (64.4)	20 (55.6)	
≥ 2b	21 (35.6)	16 (44.4)	
Treatment, n (%)			
Cytokine	12 (54.5)	10 (45.5)	0.762
Targeted therapy	32 (50.0)	32 (50.0)	0.088
Radiotherapy	7 (50.0)	7 (50.0)	0.504
Chemotherapy	5 (62.5)	3 (37.5)	0.817

As shown in Table 3, immunohistochemistry results suggested numerically differential expression of TIM-3 between the primary and metastatic tumors, with a higher detection rate in the primary than that in the metastasis (53.0% vs 45.4%). However, TIM-3 was homogeneously expressed among different metastatic sites, with no significant difference to the primary tumor except for bone metastasis, which was lower than primary tumor (χ2 = 5.98, *p* = 0.01). The expression rates of different metastatic sites were (from high to low): viscera without lung (78.6%, 11/14), ipsilateral adrenal gland (75%, 3/4), lung/lymph node (44.4%, 32/72), brain (42.1%, 8/19), others (35.0%, 7/20) and bone (39.0%, 16/41).

**Table 3:** Differential expressions of TIM-3 between the primary and metastatic tumors

	TIM-3, N(%)			*P* value
	N	Positive	Negative	
Primary	83	44 (53.0)	39 (47.0)	
Metastasis^a^	163			
Total	175	79 (45.4)	95 (54.6)	0.253
Lung/lymph node	72	32 (44.4)	40 (55.6)	0.287
Lung	24	12 (50.0)	12 (50.0)	0.795
Lymph node	46	20 (43.5)	26 (56.5)	0.300
Bone	41	16 (39.0)	25 (61.0)	0.143
Brain	19	8 (42.1)	11 (57.9)	0.391
Viscera without lung	14	11 (78.6)	3 (21.4)	0.074
Ipsilateral Adrenal gland	4	3 (75.0)	1 (25.0)	0.389
Others	20	7 (35.0)	13 (65.0)	0.148

^a^12 cases have two metastatic sites

In the paired cohort (*N* = 80), the concordance rate of TIM-3 between the primary and metastasis was 66.3% (55/83). However, TIM-3 was differentially expressed between the primary and metastasis, with a significant statistically difference (χ2 = 4.66, *p* = 0.03) and poor consistency (Kappa = 0.23, p = 0.03).

### Associations of TIM-3 expression with clinical outcomes

**Fig. 2:**
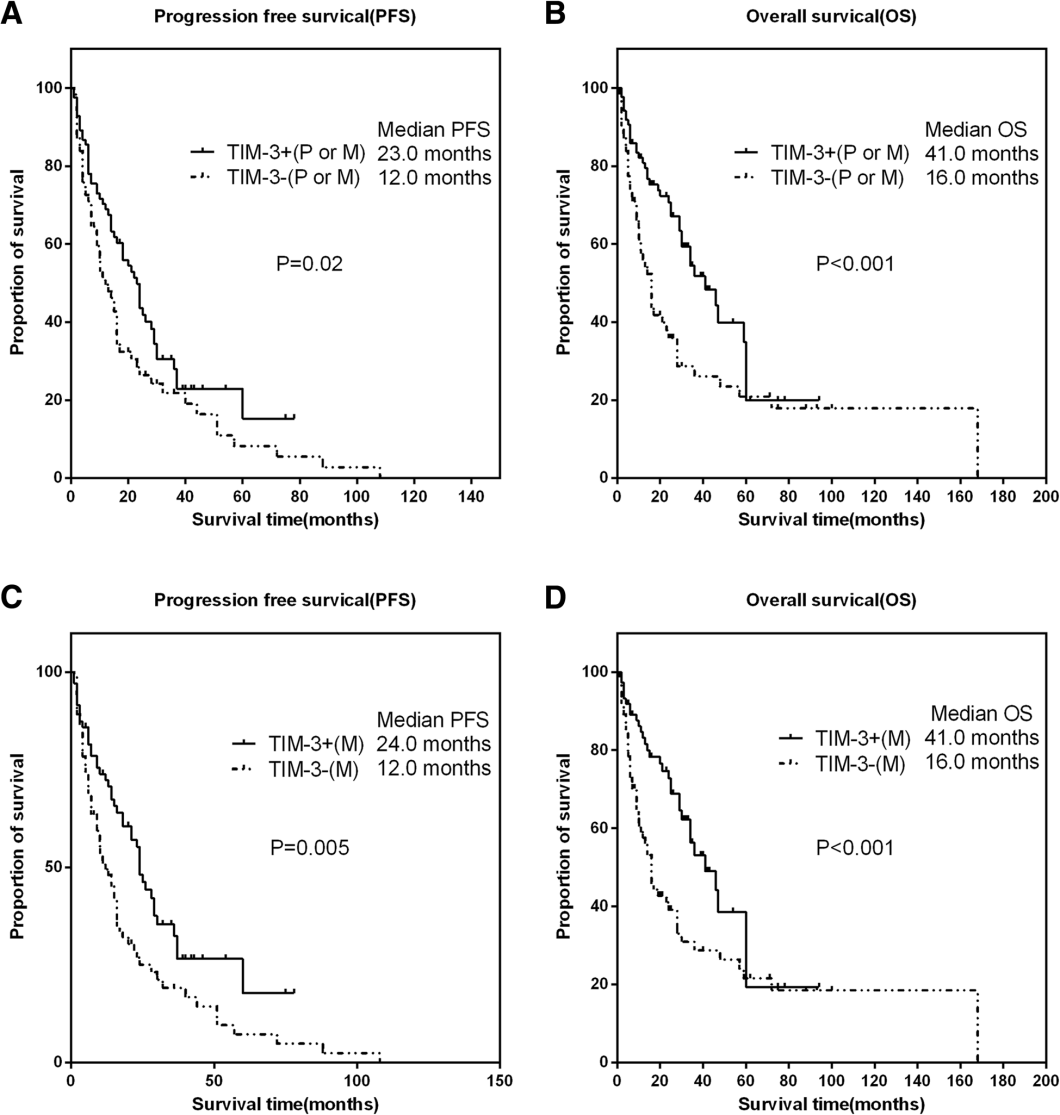
The associations of TIM-3 expressions with clinical outcomes in all patients with RCC. Representative images of kaplan-Meier survival curves of TIM-3 expression. **a** (PFS) and **b** (OS) represented TIM-3 expression either in the primary or metastatic tumor; **c** (PFS) and **d** (OS) represented TIM-3 expression in the metastatic tumor

The median PFS was 23.0 months (IQR:9.0–46.0), and the median OS was 36.0 month (IQR:16.0–70.0). In 64 patients with TKI therapy, the median PFS and OS were 21.0 and 52.0 months, respectively. Subsequent survival analysis, as shown in Fig. 2a-b, suggested that TIM-3 expression either in the primary or metastatic tumor was associated with longer PFS (HR: 0.67, 95% CI 0.45–0.99, *P* = 0.02) and OS (HR: 0.52, 95% CI 0.33–0.82, *P* < 0.001). In the metastatic tumors, patients with TIM-3 expression experienced longer PFS (HR:0.59, 95% CI 0.39–0.88, *P* = 0.005) and OS (HR: 0.51, 95% CI 0.32–0.81, *P* < 0.001) than that with negative expression (Fig. 2c-d).

**Fig. 3:**
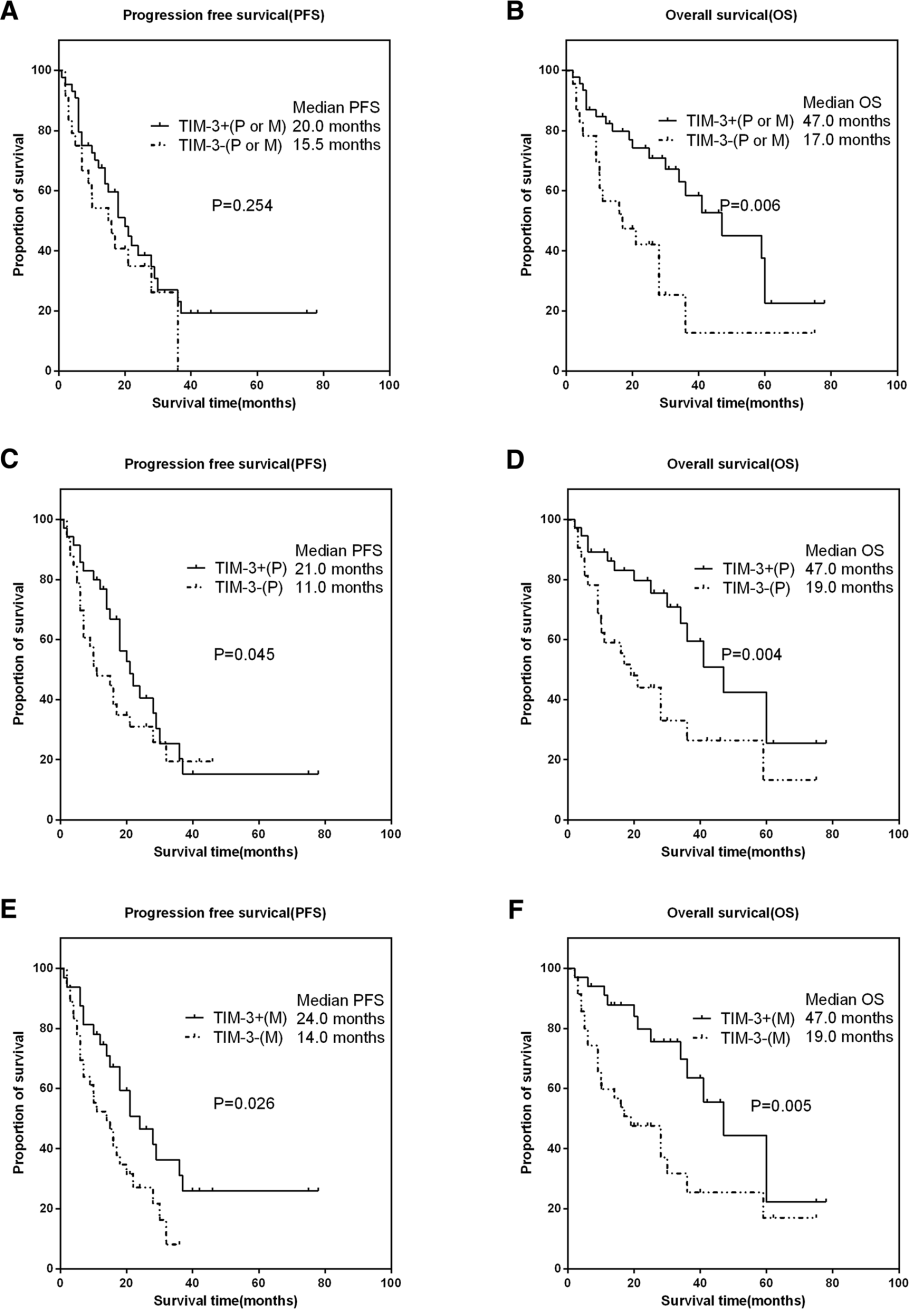
The associations of TIM-3 expressions with clinical outcomes in paired patients with RCC. Representative images of kaplan-Meier survival curves of TIM-3 expression. **a** (PFS) and **b** (OS) represented TIM-3 expression either in the primary or metastatic tumor; **c** (PFS) and **d** (OS) represented TIM-3 expression in the primary tumor; **e** (PFS) and **f** (OS) represented TIM-3 expression in the metastatic tumor

As shown in Fig. 3a-b, in the paired cohort, TIM-3 expression in the primary or metastatic tumor was not associated with PFS (HR:0.684, 95% CI 0.392–1.194, *P* = 0.254), except for OS (HR: 0.4, 95% CI 0.206–0.787, *P* = 0.006). Furthermore, TIM-3 expressions in the primary and metastatic tumors were all associated with longer PFS and OS (Fig. 3c-f).

**Fig. 4:**
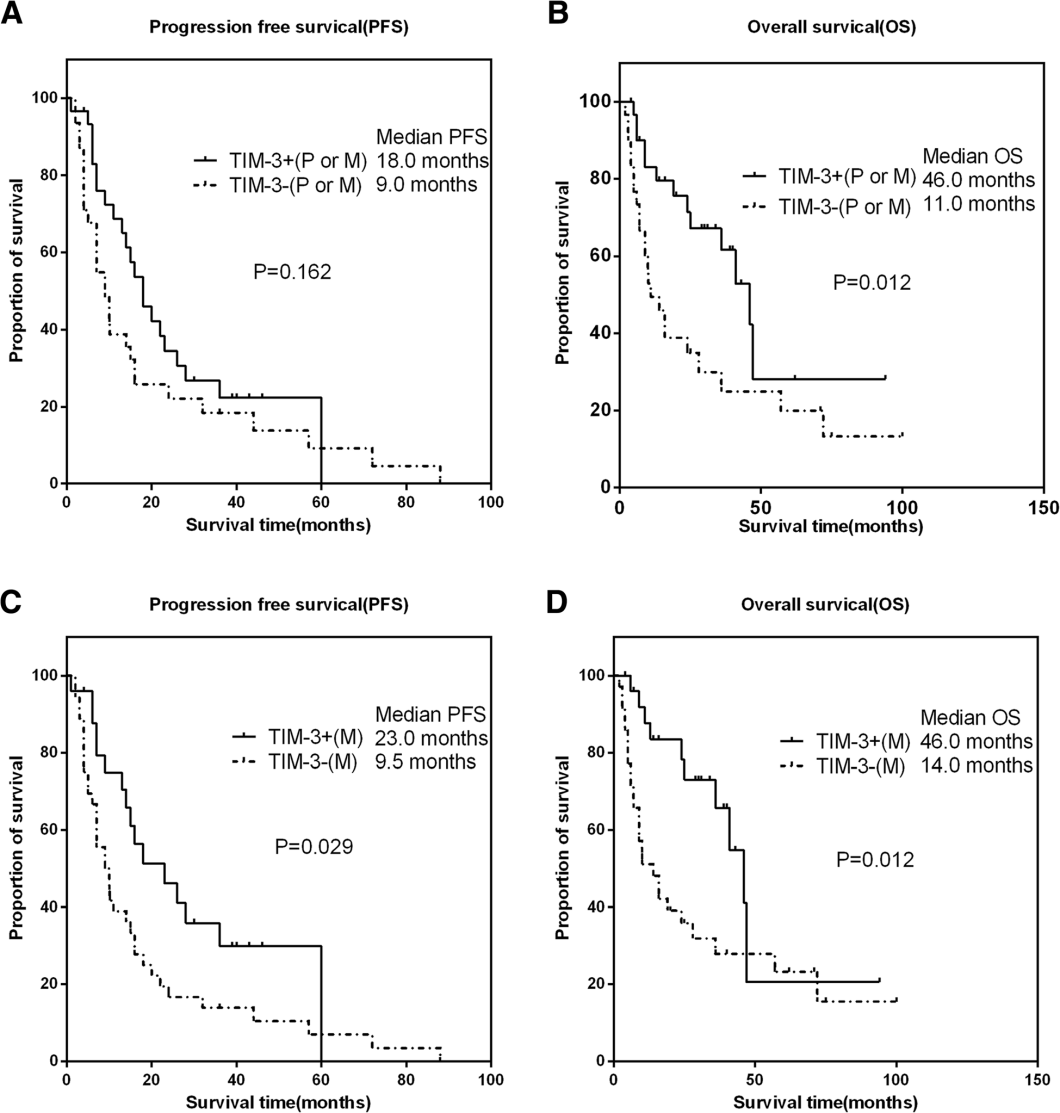
The associations of TIM-3 expressions with clinical outcomes in targeted agents treated patients with RCC. Representative images of kaplan-Meier survival curves of TIM-3 expression. **a** (PFS) and **b** (OS) represented TIM-3 expression either in the primary or metastatic tumor; **c** (PFS) and **d** (OS) represented TIM-3 expression in the metastatic tumor

Additionally, TIM-3 expression was also positively correlated with PFS and OS in patients treated with targeted agents (Fig. 4). However, as shown in Fig. 4a, TIM-3 positive either in the primary or metastatic tumor was not associated with PFS (*P* = 0.162).

### Multivariate analysis of PFS and OS in the whole, paired and targeted cohort

Unvariate analysis of PFS and OS in the three cohorts were performed (data not shown). Further multivariate analysis showed that, in the whole cohort, TIM-3 expression in metastatic tumor increased the predicted accuracy (PA) of the whole model of PFS from 74.7 to 75.6% (*P* = 0.02, Table 4). For OS, the PA of whole model was increased from 78.1 to 81.1% by adding TIM-3 expression in the metastasis (*P* = 0.005, Table 4). In the paired cohort, TIM-3 expression in metastatic tumor increased the PA of the whole models of PFS from 71.5 to 74.5% (*P* < 0.001) and OS from 78.2 to 81% (*P* = 0.015, Table 5). A same trend was also observed in patients treated with targeted agents: TIM-3 expression in the metastatic tumors significantly increased the PA values of PFS (from 73.5 to 79.1%, *P* = 0.006) and OS (from 84.2 to 87.3%, *P* = 0.009) models (Table 6).

**Table 4:** Multivariate analysis of PFS and OS in all patients (*N* = 163)

	Cox’s regression for PFS			Cox’s regression for OS		
	HR	95% Cl	*P* value	HR	95% Cl	*P* value
Gender						
Male vs < female	0.800	0.362–1.769	0.581	0.707	0.288–1.739	0.450
Age						
≥ 70 vs < 70	1.755	0.341–9.044	0.501	0.698	0.200–2.430	0.572
ISUP						
≥ 4 vs < 4	1.423	0.632–3.204	0.394	2.214	0.968–5.063	0.060
Nephrectomy						
Yes vs No	0.544	0.190–1.557	0.257	0.273	0.101–0.735	0.010
IMDC						
Low			0.956	Ref.	Ref.	0.284
Intermediate	1.067	0.374–3.040	0.904	0.396	0.123–1.276	0.121
High	1.186	0.362–3.891	0.778	0.539	0.159–1.832	0.322
T stage						
≥ T2b vs < T2b	1.512	0.688–3.319	0.303	–	–	–
CHOL (mmol/L)						
≥ 5 vs < 5	0.517	0.144–1.858	0.312	0.463	0.113–1.903	0.286
HDLC (mmol/L)						
≥ 5 vs < 5	–	–	–	0.781	0.363–1.681	0.528
LDH (IU/L)						
≥ 175 vs < 175	1.877	0.918–3.837	0.084	3.004	1.272–7.098	0.012
Na (mmol/L)						
≥ 137 vs < 137	8.458	1.909–37.468	0.005	18.993	3.629–99.415	0.001
Full model without TIM-3						
PA	0.747			0.781		
Full model with TIM-3(P or M*)						
TIM-3	0.669	0.278–1.605	0.367	0.536	0.245–1.172	0.118
PA	0.73			0.775		
Full model with TIM-3(M^#^)						
TIM-3(M)	0.377	0.162–0.876	0.023	0.351	0.156–0.787	0.011
PA	0.756			0.811		

For PFS, the *P* values for Full model with TIM-3(M) and TIM-3(P or M) compared to that without TIM-3 were 0.02 and 0.334, respectively

For OS, the *P* values for Full model with TIM-3(M) and TIM-3(P or M) compared to that without TIM-3 were 0.005 and 0.126, respectively

*P or M, primary or metastatic tumor; ^#^M, metastatic tumor

**Table 5:** Multivariate analysis of PFS and OS in paired patients (*N* = 83)

	Cox’s regression for PFS			Cox’s regression for OS		
	HR	95% Cl	*P* value	HR	95% Cl	*P* value
Age						
≥ 70 vs < 70	1.996	0.218–18.285	0.541	1.825	0.262–12.725	0.544
ISUP						
≥ 4 vs < 4	1.038	0.416–2.587	0.937	0.993	0.379–2.602	0.989
Nephrectomy						
Yes vs No	–	–	–	0.663	0.126–3.485	0.628
IMDC						
Low	Ref.	Ref.	0.451	Ref.	Ref.	0.794
Intermediate	1.242	0.443–3.480	0.680	0.704	0.214–2.317	0.564
High	1.965	0.630–6.134	0.245	0.672	0.195–2.317	0.529
T stage						
≥ 2b vs < 2b	1.565	0.714–3.432	0.264	–	–	–
ALP (IU/L)						
≥ 74 vs < 74	–	–	–	2.477	0.661–9.279	0.178
LDH (IU/L)						
≥ 175 vs < 175	1.697	0.720–4.000	0.227	3.043	0.998–9.278	0.050
Na (mmol/L)						
≥ 137 vs < 137	–	–	–	15.965	1.864–136.722	0.011
Full model without TIM-3						
PA	0.713			0.78		
Full model with TIM-3(P or M*)						
TIM-3(P or M)	1.277	0.489–3.332	0.617	0.631	0.241–1.652	0.348
PA	0.715			0.782		
Full model with TIM-3(P^#^)						
TIM-3(P)	0.953	0.413–2.2	0.911	0.594	0.237–1.486	0.265
PA	0.692			0.783		
Full model with TIM-3(M^§^)						
TIM-3(M)	0.47	0.194–1.138	0.094	0.239	0.087–0.661	0.006
PA	0.745			0.81		

For PFS, the *P* values for Full model with TIM-3(M), TIM-3(P or M) and TIM-3 (P) compared to that without TIM-3 were < 0.001, 0.126 and 0.098, respectively

For OS, the *P* values for Full model with TIM-3(M), TIM-3(P or M) and TIM-3 (P) compared to that without TIM-3 were 0.015, 0.211 and 0.206, respectively

*P or M, primary or metastatic tumor; ^#^P, primary tumor; ^§^M, metastatic tumor

**Table 6:** Multivariate analysis of PFS and OS in targeted agents treated patients (*N* = 64)

	Cox’s regression for PFS			Cox’s regression for OS		
	HR	95% Cl	*P* value	HR	95% Cl	*P* value
ISUP						
≥ 4 vs < 4	1.082	0.363–3.232	0.887	1.826	0.611–5.456	0.281
Nephrectomy						
Yes vs No	0.081	0.016–0.408	0.002	0.066	0.014–0.299	< 0.001
ECOG						
≥ 2 vs < 2	1.131	0.382–3.345	0.825	1.566	0.428–5.721	0.498
IMDC						
Low	1	Ref.	0.860	1	Ref.	0.108
Intermediate	1.195	0.300–4.760	0.800	0.302	0.055–1.646	0.166
High	1.590	0.283–8.938	0.599	1.525	0.268–8.664	0.634
Time from diagnosis to metastasis						
Synchronous vs Metachronous	0.534	0.186–1.535	0.244	–	–	–
T stage				–	–	–
≥ 2b vs <2b	3.438	0.990–11.937	0.052	–	–	–
ALP (IU/L)						
≥ 78 vs < 78	–	–	–	1.018	1.003–1.034	0.021
Na (mmol/L)						
< 137 vs ≥137	18.258	1.506–221.386	0.023	0.999	0.997–1.001	0.336
Full model without TIM-3						
PA	0.735			0.842		
Full model with TIM-3(P or M*)						
TIM-3(P or M)	0.456	0.147–1.416	0.174	0.755	0.296–1.926	0.556
PA	0.738			0.844		
Full model with TIM-3(M^#^)						
TIM-3(M)	0.167	0.048–0.586	0.005	0.537	0.226–1.277	0.159
PA	0.791			0.873		

For PFS, the *P* values for Full model with TIM-3(M) and TIM-3(P or M) compared to that without TIM-3 were 0.006 and 0.141, respectively

For OS, the *P* values for Full model with TIM-3(M) and TIM-3(P or M) compared to that without TIM-3 were 0.009 and 0.216, respectively

*P or M, primary or metastatic tumor; ^#^M, metastatic tumor

## Discussion

This was the first study that aimed to analyze the differential expressions of TIM-3 between the primary and metastatic tumors among mRCC patients. Meanwhile, based on the analyses of correlation and survival, we determined the relationship between the expression of TIM-3 and clinicopathological parameters, and its value in predicting patient’s prognosis.

Researchers have found a positive correlation between immune checkpoints (such as PD-L1) expression and treatment effect in lung cancer and melanoma [11, 12], but there was no such correlation in RCC [13]. Furthermore, published evidences have demonstrated significant differential expressions of immune checkpoints between the primary and metastatic tumors [14, 21]. TIM-3 is one of these immune checkpoints and is located in T-cell [16]. Due to the significant heterogeneity of RCC, immunological factors like TIM-3 might express differently between primary and metastatic tumors.

The expressions of TIM-3 either in the primary or metastasis suggested a better prognosis for mRCC patients in our study, especially, its expression in metastases could more accurately predict the prognosis of patients (with higher PA values both in PFS and OS models). Interestingly, the results of TIM-3 expression in predicting better prognosis of mRCC were contradictory to other tumors [22–26]. The exact reason of this phenomenon observed in mRCC remains unclear. According to our analysis about current reported studies, the possible reasons might include: 1) Most studies reported the prognostic role of TIM-3 in localized RCC, and TIM-3 may play different roles in different tumor stages; 2) Current evidences on the relationship between TIM-3 expression and prognosis are controversial among different tumor types. A study reported that TIM-3 was a protective factor in acute myeloid leukemia [20]. The same results were seen in pancreatic cancer, prostate cancer, usual vulvar intraepithelial neoplasia and colorectal cancer [27–30]. Additionally, in the ESMO 2017, Torras et al. reported that TIM-3 was differentially expressed between sunitinib refractory and sensitive groups, and was associated with benefit to sunitinib treatment in mRCC patients [31]. 3) The functional outcomes were different depending on the expressions of immune check points in tumor or tumor infiltrating immune cells. High expression of CTLA-4 on CD8 + T cells was associated with shorter OS, but TIM-3 was positively correlated with prolonged OS when expressed on CD4 + T cells [27]. 4) Different biological functions of TIM-3 might be related to different expression localization. Similar to PD-1, TIM-3 was mainly expressed in cellular membranes, but it was also expressed in the nucleus in prostate cancer and colorectal cancer [28, 30]. In bladder cancer and esophageal cancer, TIM-3 was both expressed in cellular membranes and cytoplasm [24, 32]. 5) TIM-3 also appears as a co-stimulatory signal in other non-tumor disease states [18–20].

In fact, the mechanism of TIM-3 in anti-tumor immune responses is complex. Some studies showed that TIM-3 could act as a co-stimulatory signal receptor and enhance the killing effect of cytotoxic T cells and other immune cells [17–19]. Other studies also showed that TIM-3 might act as a “rheostat,” thus orderly fine-tuning cellular responses [16]. It is unclear under what circumstances TIM-3 could appear to be co-suppressed or co-stimulatory signals, and whether co-suppression signals depend on the co-expression of other molecules still needs further validation, such as Ceacam-1 or crosstalk between different ligands of TIM-3 [16].

Multivariate analysis of the present study suggested that the expression of TIM-3 in metastases was significantly higher than that in primary tumors in predicting the prognosis of patients. It was further clarified that the expression of TIM-3 in metastatic tumors might be more indicative to patient’s prognosis. Therefore, more attention should be paid to the assessment of metastatic sites in clinical practice and research, to improve the efficacy of immunotherapy for mRCC and finally realize individualized treatment strategy.

This study was the first to evaluate the differential expression of TIM-3 in primary and metastatic RCC tumors, and is the largest study to date. However, this study still has the following limitations: 1) retrospective study design, which may induce potential selection bias; 2) limited number of paired patients; 3) different types of samples were included (frozen, resection and aspiration). Therefore, subsequent studies should focus on the observation of differences between expression and prognosis on different types of tumor.

## Conclusions

The expression differences of TIM-3 were significant between the primary and metastatic tumors. The assessment of immunological checkpoint-related protein in primary tumor might not be able to provide adequate information for clinicians to evaluate or predict the patient’s treatment-related efficacy and prognosis. The expressions of immune check points in metastatic lesions of mRCC should be given more attention, and their accurate diagnosis might be one of the effective ways to realize individual treatment. What’s more, the role of TIM-3 plays in mRCC remains to be verified.

## Abbreviations

ECOG: Eastern Cooperative Oncology Group
IHC: Immunohistochemistry
IMDC: International Metastatic Renal-Cell Carcinoma Database Consortium
mRCC: Metastatic Renal Cell Carcinoma
OS: Overall Survival
PA: Predicted Accuracy
PFS: Progression-Free Survival
RCC: Renal Cell Carcinoma
TIM-3: T cell immunoglobulin and mucin-domain containing-3
